# Current situation of H9N2 subtype avian influenza in China

**DOI:** 10.1186/s13567-017-0453-2

**Published:** 2017-09-15

**Authors:** Min Gu, Lijun Xu, Xiaoquan Wang, Xiufan Liu

**Affiliations:** 1 College of Veterinary Medicine, Yangzhou University, 48 East Wenhui Road, Yangzhou, 225009 Jiangsu China; 2 Jiangsu Co-innovation Center for Prevention and Control of Important Animal Infectious Diseases and Zoonoses, Yangzhou, 225009 Jiangsu China; 3 Jiangsu Key Laboratory of Zoonosis, Yangzhou University, Yangzhou, 225009 Jiangsu China; 4 Yangzhou Entry-Exit Inspection and Quarantine Bureau, Yangzhou, 225009 Jiangsu China

## Abstract

In China, H9N2 subtype avian influenza outbreak is firstly reported in Guangdong province in 1992. Subsequently, the disease spreads into vast majority regions nationwide and has currently become endemic there. Over vicennial genetic evolution, the viral pathogenicity and transmissibility have showed an increasing trend as year goes by, posing serious threat to poultry industry. In addition, H9N2 has demonstrated significance to public health as it could not only directly infect mankind, but also donate partial or even whole cassette of internal genes to generate novel human-lethal reassortants like H5N1, H7N9, H10N8 and H5N6 viruses. In this review, we mainly focused on the epidemiological dynamics, biological characteristics, molecular phylogeny and vaccine strategy of H9N2 subtype avian influenza virus in China to present an overview of the situation of H9N2 in China.

## Introduction

Avian influenza (AI) is initially reported in 1878 in Italy to describe the disease resulted in massive poultry death, which was then termed as “Fowl plague” to distinguish from fowl cholera in 1880 [1]. Although had being identified as filterable virus in 1901, the causative agent is formally designated as influenza A virus until 1955 [1, 2]. Apart from the highly pathogenic forms, less virulent AI viruses have been successively detected in various countries since the mid-1900s that started with the first isolate from chickens in Germany in 1949 [A/chicken/Germany/1949(H10N7)] without being recognized and defined the specific subtype till 1960. As for the H9N2 subtype, with distinguished characteristics to challenge animal industry and even human health among the low pathogenic AI forces, the protovirus is generally considered as the early isolate from turkey flocks in Wisconsin in America in 1966 [A/turkey/Wisconsin/1/1966(H9N2)] [3]. The virus spread becomes more and more extensively at about 1990s, resulting continuous viral circulation in several countries in Asia, Middle East and North Africa [4]. On one hand, H9N2 AI virus could cause damage to birds with direct pathology, coinfection and immunosuppression [5, 6]. On the other hand, H9N2 viruses not only infect mankind directly, but also provide partial or even whole set of internal genes to emerging human-lethal H5N1, H7N9, H10N8 and H5N6 reassortants [7–11], posing a substantial threat to public health. Therefore, the study of H9N2 AI virus deserves great attention.

## The etiology of AI

Avian influenza virus affiliates to the genus of type A influenza virus in the Orthomyxoviridae family, packaged with eight negative-sense and single-strand RNA segments in sequence of PB2, PB1, PA, HA, NP, NA, M and NS according to gene length [12]. Each viral gene encodes at least one protein, in which the three polymerase proteins (PB2, PB1 and PA) plus the nucleoprotein (NP) consist the minimal protein unit in forming the functional RNP structure essential for viral transcription and replication. Hemagglutinin (HA) and neuraminidase (NA) are the two major envelope glycoproteins indispensable in mediating influenza A virus to invade host cells and promoting matured newborn virions to disaggregate from cell surface, respectively [13]. Both M and NS genes utilize RNA splicing to synthesize two protein forms of matrix protein (M1) and ion channel protein (M2), nonstructural protein (NS1) and nuclear export protein (NS2), respectively. Through ribosomal frameshift, PB1 and PA genes can also be edited to generate additional PB1-F2 and PA-X proteins, effecting on virus pathogenicity [14, 15]. Based on the antigenic diversity, AI virus can be classified into 16 HA subtypes (H1–H16) and 9 NA subtypes (N1–N9), resulting in various subtype combinations. The criteria to discriminate highly pathogenic avian influenza virus (HPAIV) and low pathogenicity avian influenza virus (LPAIV) were defined at the First International Symposium on Avian Influenza in Beltsville in 1981 [1]. HPAIV only restricts to partial proportions of H5 or H7 subtype, whereas LPAIV covers all the remaining viruses. In particular, H9N2 is currently the most widely circulating and damaging LPAIV subtype in the world.

## Outbreaks and prevalence of H9N2 in China

Isolation of AI virus in China has been documented since 1970s [16]. During November 1975 to October 1979, several different subtypes of AI viruses had been isolated from imported live poultry (duck, goose, chicken) in Guangdong and Guangxi provinces, of which the most prevalent subtype is H4N6 [17, 18]. In addition, domestic scholars also described type A influenza virus from duck flocks in some meat processing enterprise in Nanjing in 1980 [19]. However, those above mentioned AI viruses were all identified from apparently healthy birds, therefore insufficient to certify the actual existence of disease outbreaks.

Till 1992, Chen et al. isolated the first H9N2 subtype LPAIV strain AID_93-1_ (once erroneously identified as H9N3 subtype then), also the earliest published report of AI outbreaks in mainland China [20]. During November 1992 to May 1994, a total of 17 chicken farms and two minor poultry farms had suffered from AI outbreaks in regions of Guangdong province [20, 21]. A few years afterwards, several other parts in China intermittently reported sporadic disease outbreaks caused by H9N2 [22–24]. However, a massive H9N2 epizootics occurred from fall to winter in 1998, initially starting from Hebei province and rapidly spreading to majority of poultry raising areas nationwide in only 2 months [25, 26]. According to the statistics, the ratio of chicken flocks subjected to H9N2 subtype AI infection accounted for 93.89% in the period of 1996–2000, thereby demonstrating that H9N2 was the predominant subtype affecting poultry farming from the end of twentieth century to the beginning of twenty first century [27]. Even to this day, H9N2 is still one of the three primary AI subtypes devastating poultry industry other than the notorious H5N1 and emerging rookie of H7N9.

Theoretically, emerging diseases could possibly be effectively controlled by a stamping-out policy before disseminating into vast areas [28]. However, the optimal eradication opportunity for H9N2 through timely culling of infected poultry was missed during 1992–1998 in China, as the disease has remarkably spread into large regions especially since 1998 and the vaccination strategy has been extensively executed since then [29]. Presently, H9N2 has become stably established in chicken flocks to acquire the endemicity in vast majority of China, accompanied with the substantial implementation of vaccination programs [5]. Moreover, the virus is yet prevalent in wild birds, live poultry markets, backyard flocks and environment [30, 31]. Generally, the inherited complex breeding and trading patterns of poultry industry contributed critically to the current epidemiological situation of H9N2 in China. On one hand, traditional small-scale and backyard-level raisings such as free ranging and mixed ranging still occupy certain ratio in poultry production nationwide, while their biosecurity condition and vaccination coverage are relatively unsatisfactory as compared with typical intensive operations. On the other hand, live poultry markets (LPMs) as a distinctive manifestation of the consumption style that freshly-killed poultry meat is much more preferred rather than chilled or frozen meat, has provided a tremendous gene pool of avian influenza viruses which is evidenced by the continued high virus detection rate including multiple HA/NA subtypes [32]. It is worth noting that interventions involving implementation of one or two rest days per month in the wholesale and retail LPMs could significantly reduce the H9N2 isolation rates [33]. As China is still located on the important flyways for migration, the huge amount of domestic waterfowls which frequently contact the ecointerface with wild waterfowls when sharing common water or makeshift inhabitance also facilitated the persistence and evolution of H9N2 viruses in environment by means like inter-transmission and gene reassortment between birds [30]. Ecologically, at least those above mentioned intricate factors jointly shaped the enzootic status of H9N2 in China.

## Genetic evolution of H9N2

H9N2 subtype AI virus is extensively distributed worldwide, generally divided into two major lineages of North-American lineage and Eurasian lineage. Specifically, the Eurasian lineage further blooms into various virus clusters, as represented by A/chicken/Beijing/1/1994(BJ/94-like) or A/duck/Hong Kong/Y280/1997(Y280-like), A/quail/Hong Kong/G1/1997(G1-like), A/duck/Hong Kong/Y439/1997(Y439-like), A/chicken/Shanghai/F/1998(F/98-like) and so on [34–36]. Comparing with the H9N2 viruses in Central Asia and the Middle East, Chinese isolates clustered independently as referred from the phylogenetic trees of HA and NA genes [36]. In China, G1-like circulated mainly in quails is of geography superiority in southern regions, whereas BJ/94-like and F/98-like prevailed in chicken flocks are regnant in northern and eastern areas, respectively [26, 35].

### HA phylogenetic clades

To further systematically understand the evolutionary dynamics of H9N2 subtype AI virus globally, four stem evolutionary clades of h9.1–h9.4 have been designated by Jiang et al. to map the HA gene phylogeny through comparing more than 1000 HA sequences retrieved from GenBank, as referred to the nomenclature of the Asian H5N1 HPAIV defined by the WHO/OIE/FAO H5N1 working group [37, 38]. Particularly, h9.1 and h9.2 just corresponded to early North-American isolates in 1966 and the nineties, respectively. H9.3 covered the widest temporal span including Asia, Europe, Africa, Pacific and North America, so did expand the longest spatial range from 1976 until now. The most vast clade h9.4 included two subclades of h9.4.1 and h9.4.2, which coordinated to the G1-like (h9.4.1.1) and Y280-like (h9.4.2.4) H9N2 viruses prevailing in most Asian countries ever since 1994, respectively. In more detail, h9.4.1 contained isolates from Pakistan, India, Iran and Israel, whereas h9.4.2 accommodated exclusively Chinese strains. Chronologically, domestic H9N2 viruses before 2007 generally belonged to clades h9.4.2.1–h9.4.2.4, in which h9.4.2.1 equaled to the above mentioned F/98-like viruses. Thereafter, h9.4.2.5 represented by A/chicken/Guangxi/55/2005(H9N2) has become predominant step by step, whilst h9.4.2.6 distinguished by A/chicken/Guangdong/FZH/2011(H9N2) mainly in southern China has also acquired establishment and tended to spread readily across the country from about 2010. Hence, currently, h9.4.2.5 and h9.4.2.6 have co-circulated in China, while of which the former H9N2 viruses are yet superior over the latter ones.

### Genotypic diversity

**Figure 1:**
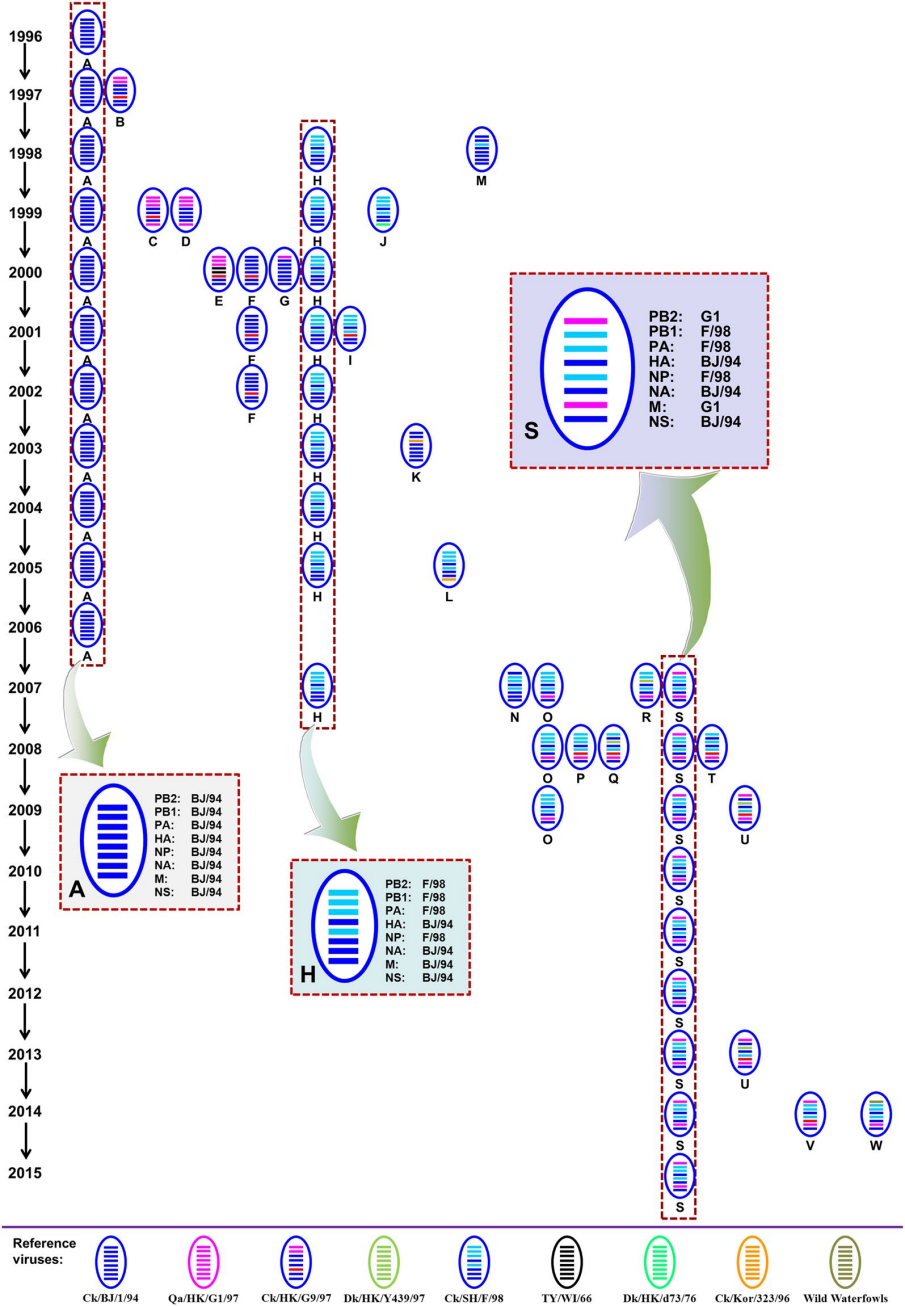
**Genotypic diversity of H9N2 subtype avian influenza viruses in China during 1996–2015.** The eight horizontal bars in oval (from top to bottom) represent PB2, PB1, PA, HA, NP, NA, M and NS genes, respectively. Each color represents a virus lineage. The resulting genotype designation is depicted below.

Owing to the segmented nature of AI virus genome, when two or more virus strains concurrently infect a single cell, exchange of gene segments would occur among different virus particles via gene reassortment to generate a series of newborn viral descendants inheriting parental components. Certainly, H9N2 subtype AI virus is also without exception that distinct virus clusters could reassort with each other or with other AI subtypes to produce various genotypes, which is defined on the basis of the combination of each individual gene phylogenies. For instance, virus harbored all the gene constellation from BJ/94-like is designated as genotype A, variant of three polymerase genes and NP gene from F/98-like while the remaining four genes from BJ/94-like is assigned for genotype H. Thus far, H9N2 subtype AI virus in China has evolved into diversified clusters and genotypes (A–W), showing clear spatio-temporal divergence (Table 1; Figure 1) [7, 26, 29, 39, 40]. Among the rest, three major genotypes of H9N2 subtype AI virus containing A, H and S, have predominated in chicken flocks during different periods since the nineties [6, 7, 29, 41]. In particular, early genotype A prevailing in the nineties had gradually been replaced by genotype H, evident of better adaptation in poultry and easier reassortment with other AI viruses, after 2000 [6]. However, genotype S with exogenous G1-like PB2 and M genes on the genetic backbone of F/98-like viruses emerged around 2007 and had become increasingly established in chickens afterwards, especially in the Yangtze River Delta region in eastern China [6, 7]. Updated epidemiological studies in more recent years also suggest the supreme of genotype S there [40]. Consistently, the additionally categorized genotype G57 (generally equivalent to genotype S) demonstrated greater infectivity than the other genotypes, and had been dominating ever since 2010 across China to cause severe damages to poultry farming [42].

**Table 1:** **Genotypes of H9N2 subtype avian influenza viruses in China.**

Genotype	Emerged year	Gene constellation								References
		PB2	PB1	PA	HA	NP	NA	M	NS	
**A**	**1994**	**BJ/94**	**BJ/94**	**BJ/94**	**BJ/94**	**BJ/94**	**BJ/94**	**BJ/94**	**BJ/94**	[29]
B	1997	G1/97	G1/97	BJ/94	BJ/94	BJ/94	G9/97	BJ/94	BJ/94	
C	1999	G1/97	G1/97	G1/97	BJ/94	BJ/94	G9/97	BJ/94	G1/97	
D	1999	G1/97	G1/97	G1/97	BJ/94	BJ/94	BJ/94	BJ/94	G1/97	
E	2000	G1/97	G1/97	G1/97	TY/WI/66	TY/WI/66	G9/97	BJ/94	BJ/94	
F	2000	BJ/94	BJ/94	BJ/94	BJ/94	BJ/94	G9/97	BJ/94	BJ/94	
G	2000	G1/97	BJ/94	BJ/94	BJ/94	BJ/94	BJ/94	BJ/94	BJ/94	
**H**	**1998**	**F/98**	**F/98**	**F/98**	**BJ/94**	**F/98**	**BJ/94**	**BJ/94**	**BJ/94**	
I	2001	F/98	F/98	F/98	BJ/94	F/98	G9/97	BJ/94	BJ/94	
J	1999	F/98	F/98	F/98	BJ/94	F/98	BJ/94	BJ/94	d73/76	[26]
K	2003	BJ/94	BJ/94	Kor/323/96	BJ/94	BJ/94	BJ/94	BJ/94	BJ/94	
L	2005	F/98	F/98	F/98	BJ/94	F/98	BJ/94	BJ/94	Kor/323/96	
M	1998	BJ/94	BJ/94	F/98	BJ/94	BJ/94	BJ/94	BJ/94	BJ/94	[39]
N	2007	BJ/94	F/98	F/98	BJ/94	F/98	BJ/94	BJ/94	BJ/94	
O	2007	F/98	F/98	F/98	BJ/94	F/98	BJ/94	G1/97	BJ/94	
P	2008	F/98	F/98	F/98	BJ/94	F/98	G9/97	G1/97	BJ/94	
Q	2008	F/98	BJ/94	Y439/97	BJ/94	F/98	G9/97	G1/97	BJ/94	
R	2007	F/98	F/98	Y439/97	BJ/94	F/98	BJ/94	G1/97	BJ/94	[7]
**S**	**2007**	**G1/97**	**F/98**	**F/98**	**BJ/94**	**F/98**	**BJ/94**	**G1/97**	**BJ/94**	
T	2008	F/98	BJ/94	F/98	BJ/94	F/98	G9/97	G1/97	BJ/94	
U	2009	G1/97	BJ/94	Y439/97	BJ/94	F/98	G9/97	G1/97	BJ/94	
V	2014	G1/97	F/98	F/98	BJ/94	F/98	G9/97	G1/97	BJ/94	[40]
W	2014	Wild Waterfowls	F/98	F/98	BJ/94	F/98	F/98	G1/97	BJ/94	

Genotypes were defined according to the array mode of the eight gene phylogenies, those which have or had persisted for a long time in China are labeled in bold

BJ/94: A/chicken/Beijing/1/1994(H9N2)-like; G1/97: A/quail/Hong Kong/G1/1997(H9N2)-like; G9/97: A/chicken/Hong Kong/G9/1997(H9N2)-like; Y439/97: A/duck/Hong Kong/Y439/1997(H9N2)-like; TY/WI/66: A/turkey/Wisconsin/1/1966(H9N2)-like; F/98: A/chicken/Shanghai/F/1998(H9N2)-like; Kor/323/96: A/chicken/Korea/38349-p96323/1996(H9N2)-like; d73/76: A/duck/Hong Kong/d73/1976(H6N1)-like

## Biological property variation of H9N2

The premier isolates of H9N2 just infected turkeys, rarely encroached on chickens, but have gradually adapted to chickens and acquired pathogenicity after years of evolution [1, 3]. Since the initial isolation of H9N2 virus in China, its host range and virulence have become increasingly wider and stronger, respectively [5, 26, 35, 42, 43]. As revealed by a continuous surveillance on H9N2 subtype AI virus in eastern China from 1999 to 2008, most viruses before 2000 were competent to propagate in inoculated chickens but inadequate to be transmissible through respiratory droplets [26]. In contrast, variants after 2001 not only replicated well in vivo but also transmitted efficiently by respiratory droplets in chickens [44]. Comparing with the ones prior to 2010, H9N2 isolates circulating during 2010–2013 showed an obviously higher isolation rate and titers, as well as a longer period of virus shedding especially from cloaca in challenged chickens [42]. It was recently demonstrated that such improved viral fitness was resulted from the substitution of BJ/94-like M gene with the G1-like [45]. Specifically, H9N2 viruses containing G1-like M gene not only exhibited significantly efficient early augment of viral mRNA and vRNA to increase the amount of produced protein and benefit the release of progeny virions, but also conferred extrapulmonary virus spread in chickens [45]. Moreover, characterization of H9N2 viruses ranging from 2009 to 2013 in southern China indicated that natural H9N2 isolates of chicken origin had gradually acquired the preference for human-type α-2,6 sialic acid receptors, and several variants even developed the airborne transmissibility in ferrets [46].

## Internal gene cassette reassortment of H9N2

**Figure 2:**
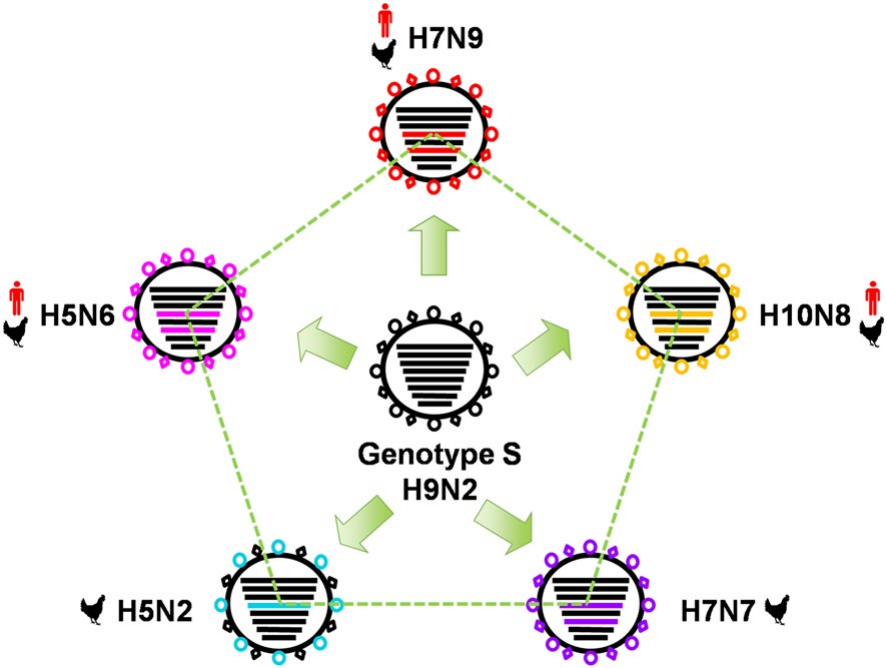
**Illustrative scheme of the events induced by reassortment of whole set of internal genes from H9N2 to generate novel influenza A reassortants.** The eight horizontal bars in circle (from top to bottom) represent PB2, PB1, PA, HA, NP, NA, M and NS genes, respectively. The ring and prismatic shape on outer surface of circle represent the HA and NA proteins, respectively. Each color represents a specific virus subtype. The black poultry label indicates that the generated influenza A virus challenges birds and the red figure label indicates that the reassortant can also infect human beings.

It is acknowledged that the variation mechanism of AI virus mainly includes antigenic drift and genetic shift, with the former featured by point mutation of key amino acids in major immunoprotective proteins whereas the latter resulted from genomic reassortment [47]. As compared with genetic drift, gene reassortment poses a more radical effect on influenza virus by generating totally brand-new viruses with competitive advantage to spread widely such as those causing influenza pandemics in history [48–51]. According to literatures, H9N2 not merely donate partial gene segments but also the whole set of internal genes to reassort with other influenza A viruses [52, 53]. Especially in the past few years, the phenomenon that the six internal genes of H9N2 constituting a relatively stable community to transfer into other emerging reassortants as a whole cassette seems more distinguished (Figure 2). For example, the newly detected chicken H7N7 viruses in Wenzhou city of Zhejiang province, the human-infecting H7N9 and H10N8 viruses initially reported in 2013, and the more recent clade 2.3.4.4 human-lethal H5N6 viruses, were all generated on the basis of complete internal genes from H9N2 subtype AI viruses [9, 10, 54–56]. In addition, H9N2 even dedicated all the other seven gene segments except HA to the clade 7.2 HPAI H5N2 natural reassortants in recent years [57, 58]. Despite diversity, those H9N2 donor viruses all pertain to the unique S genotype prevailing in chicken flocks in China since 2007 [7]. As influenza A virus proved to choose gene segments specifically for package when more than one kind of viruses co-infect the same host cell, whether the intrinsic vRNA–vRNA interaction contributed crucially to the molecular mechanism of this particular internal-gene-cassette re-assortment deserves further exploration [59–61].

## Vaccine strategy for control of H9N2

Presently, H9N2 subtype AI virus has been widely spread in China, and has established stable lineages in commercial chicken flocks with endemicity [40]. Despite that the mortality caused by H9N2 generally not exceed 20%, it usually leads to respiratory and egg-drop symptom, as well as sever secondary infection of other respiratory diseases, affecting poultry productivity [5, 29]. Therefore, at current stage, vaccination is still one of the principle strategies to control H9N2 AI in China apart from biosecurity.

### Conventional whole-virus inactivated vaccines

The majority of commonly used AI vaccines are killed whole-virus vaccines, prepared from formaldehyde inactivation of virus-containing allantoic fluids proliferated via chicken embryos and accompanied with adjuvants, manifesting favorable immune efficacy [62]. Domestically, various H9N2 strains have been used for inactivated vaccine development, wherein the F strain and SS strain respectively belonging to genotype H and genotype A are the two typical representatives [63, 64]. The F strain is a natural reassortant of chicken origin isolating from Shanghai in 1998, with its polymerase genes being replaced with counterpart gene segments from distinct H9N2 clusters in ducks. This F/98-like virus entirety had existed over a long time in chicken flocks in China, and even served as the donor to provide internal genes for further reassortants until recently [65, 66]. As for SS vaccine, it is developed from the seed isolated from Guangdong province in 1994, which is also the first commercial vaccine for control of H9N2 subtype AI in China. However, as the ongoing evolution of H9N2 viruses, vaccination failure due to infection with prevailing antigenic variants evidently challenges the efficacy of the vaccines in China, like that in many other countries such as Iran and Korea [40, 67–72]. Therefore, updated vaccine seed strains based on continuous surveillance data have gradually been preparing and permitting for clinical practice. To simplify the immune procedure to reach an ideal goal of “one injection preventing multiple diseases”, a massive of double or multiple combined vaccines have been designed such as the triplex inactivated vaccines simultaneously against AI (H9 subtype), Newcastle disease and infectious bronchitis [73].

### Recombinant and vector virus vaccines

Inactivated whole virus vaccine mainly elicits humoral immune response, deficient in inducing effective mucosal and cellular immunity. Furthermore, it also interferes with immunological surveillance and epidemiological investigation of AI virus under the condition of current technology. Therefore, novel DIVA (differentiating infected and vaccinated animals) vaccines against H9N2 come into being, including recombinant live virus vectored vaccine, subunit vaccine, DNA vaccine, VLPs (virus like particles) vaccine and so on. They could supplement certain shortages of traditional vaccines and are popular for AI vaccine development nowadays. Frequently used live virus vectors contain recombinant fowlpox virus, Newcastle disease virus, Marker’s disease virus, etc [74–76]. Subunit vaccine is generally developed based on the extraction of immunogenic proteins (usually HA) of AI virus, without introducing viral particles. Large amounts of HA protein could be acquired by ligation of HA gene with expressing plasmid vector for amplification, such as in the baculovirus expression system [77]. As for DNA vaccine, the exogenous gene encoding for protective antigen is initially cloned to eukaryotic expressing vector, followed by administrating the constructed DNA plasmids into animals to get expressed in vivo and to stimulate specific humoral and cellular immunity [78]. VLPs are self-assembled hollow protein particles by one or more viral structural proteins, containing no viral genetic materials but resembling integral viruses in appearance. Despite without infectivity, VLPs could still retain immunogenicity to provoke effective immune response and to serve as safe vaccines [79]. So far, a great number of novel genetically engineered AI vaccines have been designed in China, however, many of which are still in the stage of technical research and reserve, immature for clinical usage yet.

## Interspecies transmission of H9N2

### H9N2 in pigs

Apart from various kinds of poultry, H9N2 subtype AI viruses could also infect pigs, the long considered mixing vessel for mammalian and avian influenza variants. It is revealed by epidemiological survey that H9N2 viruses were isolated from pigs naturally when transported from southern China to Hong Kong for sale, as early as in 1998 [80]. Subsequently during 2001–2008, H9N2 had been detected incessantly in swine herds in several provinces covering Shandong, Fujian, Henan, Jiangxi, Guangdong, Guangxi, Hebei and so on [81–84]. In addition, the identified swine H9N2 isolates exhibited evident genetic and antigenic complexity with diversified genotypes [85]. Serological investigation also manifested the infection of H9N2 viruses in Chinese pig population [86–88].

### H9N2 in humans

What’s more noteworthy, H9N2 subtype AI viruses have already acquired the ability to break through species barrier and directly invade human beings without intermediate hosts. The first documentation of human-infecting H9N2 viruses in China traced back to 1998, as described that five H9N2 strains were cultured from laryngopharyngeal mucus of flu-like outpatients and inpatients in southern regions [89]. Further gene sequence analysis indicated that those H9N2 human isolates probably derived from local chicken flocks [90]. In March 1999 in Hong Kong, another two children were confirmed infection with H9N2 viruses, with their genomic sequences highly homologous with the quail strain A/quail/Hong Kong/G1/1997 [91, 92]. Therefore, quails had also been suggest to play important roles in cross-species transmission of H9N2 viruses [93]. Still in 1999, A/chicken/Hong Kong/G9/1997-like H9N2 virus repeatedly isolated from human population in November in southern China [94]. Again in December 2003, Hong Kong reported a second human infection event of H9N2 virus, of which all the eight gene segments were of avian origin and clustered most intimately with those extensively distributed in live poultry market there [95]. Yet recently, laboratory-confirmed human infection of H9N2 virus have continuously been reporting sporadically from WHO, with an apparently higher rate in the last few years and even one fatal case additionally suffering from chronic underlying conditions in 2016 [45]. Besides, quite a number of people prove to have been exposed to H9N2 viruses by serological data, especially those poultry workers [89, 96–98]. Distinct from HPAI H5N1 infection, the overall human symptoms induced by H9N2 are analogous to seasonal flu with rapid recovery and no lethality. However, just such mild infection has made H9N2 easily be negligible in clinical, facilitating to adapt further in the body by reassortment with other human influenza viruses to yield potential variants with high reproductivity and even efficient interpersonal transmissibility.

## Conclusion

Although being classified as LPAIV, H9N2 subtype AI virus is extensively distributed in chicken flocks to pose a persistent challenge. In China, traditional raising system of livestock including free-ranging and polyculture, continuously occupies a crucial status yet. It is inevitable for chicken to contact with domestic or wild waterfowl, which harbored large amount of H9N2 viruses. These apparently healthy latent birds could serve as the “Trojan horses” in chicken flocks to cause the circulation of H9N2. Furthermore, the LPMs extending throughout China still played an indispensable role in hosting and disseminating of H9N2 AI virus, as evidenced by significant higher rates of virus isolation than other locations. However, focusing on LPMs management, innovative control measures targeting principally against the emerging avian influenza A(H7N9) virus such as closure of LPMs or other more sustainable but yet effective interventions including washing and cleaning once a day, disinfecting once a week, having rest days once a month and banning live poultry overnight, as well as separating of aquatic and non-aquatic live poultry, would certainly simultaneously reduce the risk of H9N2 contamination at source and deserve high priority in implementation. On account of incessant viral mutation and reassortment, natural variants with increased pathogenicity have been emerging periodically. Even though vaccination remains one of the primary strategies to control H9N2 subtype AI in China, the majority of vaccine recipients are actually still under siege of wild-type variants. Therefore, disease outbreaks would still occur in vaccinated flocks in case of descended protection level or different kinds of immune failure. It is more prior important to establish favorable biosecurity management and take all practicable measures to control infection source, preventing virulent variants from intruding the poultry flocks. On the other hand, the human-infecting events of H9N2 AI virus deserve to be treated scientifically and rationally. Once animal influenza is controlled, should the risk of emerging human pandemic influenza be decreased to minimum level.

## Abbreviations

AI: avian influenza
PB2: polymerase basic protein 2
PB1: polymerase basic protein 1
PA: polymerase acidic protein
HA: hemagglutinin
NP: nucleoprotein
NA: neuraminidase
M: matrix protein
NS: nonstructural protein
HPAIV: highly pathogenic avian influenza virus
LPAIV: low pathogenicity avian influenza virus
LPMs: live poultry markets
WHO: World Health Organization
OIE: World Organisation for Animal Health
FAO: Food and Agriculture Organization of United Nations
BJ/94: A/chicken/Beijing/1/1994(H9N2)
F/98: A/chicken/Shanghai/F/1998(H9N2)
G1: A/quail/Hong Kong/G1/1997(H9N2)
SA: sialic acid
DIVA: differentiating infected and vaccinated animals
VLPs: virus like particles
